# Systematic underestimation of polycyclic aromatic hydrocarbon aqueous concentrations in rivers

**DOI:** 10.1007/s11356-024-33787-9

**Published:** 2024-05-25

**Authors:** Ottavia Zoboli, Nikolaus Weber, Katharina Braun, Jörg Krampe, Matthias Zessner

**Affiliations:** 1 Institute for Water Quality and Resource Management, TU Wien, Karlsplatz 13, 1040 Vienna, Austria; 2 Environment Agency Austria, Spittelauer Lände 5, 1090 Vienna, Austria

**Keywords:** Suspended solids, Suspended particulate matter, Riverine load, PAH, Extraction

## Abstract

Polycyclic aromatic hydrocarbons (PAHs) are a widespread group of organic contaminants whose presence in water bodies is cause of severe concern. With few exceptions, the majority of PAHs is hydrophobic, presents a high adsorption affinity, and is thus primarily transported within river systems during high-flow events together with suspended particulate matter (SPM). Evidence exists of analytical challenges related to the incomplete extraction of PAHs adsorbed to solids and thus to a potential negative bias in the chemical analysis of PAHs in bulk water samples with high SPM content. Despite this, partly due to the elevated efforts required to collect representative samples containing sufficient SPM for the separate PAH analysis in this matrix, several investigations rely on the analysis of aqueous samples. This study tests the hypothesis that surveys based exclusively on bulk water may lead to a systematic underestimation of the real contamination level and transport of PAHs in rivers. Six high-turbidity events were examined in three Austrian rivers applying time-integrated sampling and simultaneously analyzing PAHs in total bulk water, filtered water, SPM, and supernatant. Despite an unavoidable degree of uncertainty in such challenging sampling scheme, the results indicate that measurements performed with best available standard methods in bulk water samples determined in average only about 40% of the theoretically expected total PAHs concentrations derived from the analyses in SPM. Such deviation has important implications for the reliable assessment of the compliance with environmental quality standards as well as for surveys aimed to estimate riverine loads, validate emission models, and understand the transport dynamics of PAHs in rivers. Whereas the first objective, e.g., in European countries, is alternatively achieved via monitoring in biota, the latter ones require efforts directed to complement monitoring campaigns with separate sampling of SPM, with monitoring of suspended solids transport to appropriately select and interpret the results of water samples and to improve the chemical analysis of PAHs in bulk water samples with high solids content.

**Supplementary Information:**

The online version contains supplementary material available at 10.1007/s11356-024-33787-9.

## Introduction

Polycyclic aromatic hydrocarbons (PAHs) are a group of organic compounds that, in addition to being released by natural geogenic sources and wildfires, enter the environment primarily through anthropogenic activities such as the combustion of coal, oil, gas, and biomass, or the use of bitumen and petroleum-based products (Cheruiyot et al. 2015). Once released into the environment, they reach surface waters predominantly via stormwater and combined sewer overflows, direct atmospheric deposition, and soil erosion (Amann et al. 2019). Their occurrence in water bodies is cause of concern, as they are persistent, bioaccumulating, and toxic. Eight PAHs are on the European Union (EU) list of priority substances (EU 2013), with six of them being classified as priority hazardous substances. According to the EU Water Framework Directive (WFD, 2000/60/EEC), for such substances, the Environmental Quality Standards (EQS) defined in surface waters by EU (2013) shall not be exceeded and discharges, emissions and losses must be ceased or phased out.

With the exception of PAHs with low molecular weight such as naphthalene, the majority of these substances is hydrophobic and presents a high adsorption affinity. It is thus predominantly during high-flow and high-turbidity events that the transport in fluvial systems occurs, when the increased hydraulic energy mobilizes contaminated soil and sediment particles (Patrolecco et al. 2010; Sicre et al. 2008).

Due to the fact that EQS are defined for total — or bulk — water and biota, official monitoring campaigns in Europe typically focus on these two matrices, although the assessment of the chemical status is predominantly based on the analysis of biota. Further, scientific studies aimed at better understanding the particle-bound transport of PAHs and its dynamics in rivers often rely on the PAH analysis in bulk water samples (Glaser et al. 2020; Rügner et al. 2019; Schwientek et al. 2017). Such works build on the conceptual approach developed by Schwientek et al. (2013), which derives the particulate fraction of PAH concentration in water as the slope of the linear regression curve between the total PAH concentration in bulk water samples and their total suspended solids (TSS) content.

However, various analytical investigations pointed out the existence of critical issues related with the analysis of PAHs in bulk water samples with elevated TSS content. According to Ademollo et al. (2012) and Coquery et al. (2005), the problem of the liquid–liquid extraction step in the analysis of bulk water is the poor recovery of analytes in the presence of particulate and/or dissolved organic carbon, as the organic solvent cannot effectively penetrate the particles or microaggregates surrounded by water. Although validation studies with complex matrices (e.g., wastewater and sewage sludge samples) have shown that liquid–liquid extraction recovery was satisfactory even for hydrophobic compounds such as PAHs (ISO 2005), Ademollo et al. (2012) question the reliability of such conclusions, arguing that the spiking method used to assess recovery is not sufficiently clear and robust. Similar problems of incomplete extraction of hydrophobic organics adsorbed on particles or of their adsorption on the surfaces of the containers used in this processing step have been identified in several studies not only for liquid–liquid extraction but also for solid-phase extraction (Vignati et al. 2009; Jeanneau et al. 2007; Coquery et al. 2005). The risk of a negative bias in the results was also addressed by Busch et al. (2007), who investigated in selected German rivers the influence of sampling and sample preparation on the results in the determination of different priority substances. As part of that study, a special investigation showed that the PAHs concentrations found in the filter residue for the PAHs with higher molecular weight significantly exceeded the PAHs content determined in the total water sample. The authors’ explanation was that the particulate-bound PAHs could be extracted only incompletely from the original sample via the extraction procedure. According to Busch et al. (2007), earlier investigations conducted by the State Environmental Agency of North Rhine-Westphalia showed that only about 40–60% of the particulate-bound fraction was detected in the liquid–liquid extraction with n-hexane according to EN ISO 17993. A further indication of potential underestimation of real PAHs concentrations in rivers stems from the contradictory results obtained in different monitoring campaigns conducted in Austria. Whereas a targeted sampling of suspended particulate matter (SPM) revealed concentrations of most PAHs in the approximate range of 10–60 μg kg^−1^ dry matter (dm) (Zoboli et al. 2019), a subsequent official national survey did not confirm the expected higher PAHs concentrations in the bulk water samples with TSS content above 100 mg l^−1^ (GZÜV 2013). A similar contradiction was revealed by Chiffre et al. (2015), who found that the water quality status assessment based on water samples in two French rivers would lead to a positive outcome, whereas the evaluation based on SPM and sediments pointed to the exceedance of available guidelines. The cause of the difference was however not explored.

This apparent discrepancy is highly relevant for the reliability of the assessment of compliance with EQS where an appropriate or sufficient amount of biota is not available. It is also important for estimating accurately riverine loads of PAHs. These in turn are essential for establishing emission inventories and validating models used for risk assessment and for the evaluation of the effectiveness of emission control measures (EC 2012).

This study presents the findings of a targeted survey designed to investigate the aforementioned inconsistency in more detail. The core idea was to sample bulk water and SPM during specific turbidity events in rivers, separately but simultaneously. The initial specific objective was to obtain a sufficient quantity of SPM to enable the accurate analysis of PAHs in this matrix for each individual event. This would subsequently allow to calculate the theoretical total concentration of PAHs in bulk water by combining the PAHs measured values in SPM with the TSS content of bulk water and with the PAHs measured values in filtered water (obtained from the bulk water samples). The purpose of this experimental setup was to test the hypothesis that the measured concentrations of PAHs in bulk water would be significantly lower than the theoretical calculated concentrations. By doing so, we aimed to test the overarching hypothesis that surveys exclusively relying on bulk water samples may lead to a systematic underestimation of the real contamination level and transport of PAHs in rivers.

## Materials and methods

### Sampling

Six high-turbidity events were sampled in three rivers in Austria with a combination of time-integrated techniques and devices that enabled the near-simultaneous PAHs analysis in bulk and filtered water samples, in SPM samples and in the supernatant water after SPM decantation. The criterion to select the events was an increased TSS transport greater than at least 200 mg l^−1^. The sampling took place between May 2022 and April 2023 at rivers located in the South-Eastern region of Austria, at sites in which the authors operate water quality monitoring stations. Such stations are placed next to governmental hydrological gauges and are equipped, among others, with sensors for the online measurement of turbidity and water level and with remotely controlled autosamplers.

Wulka is a river with a catchment size of approximately 400 km^2^. The terrain is rather flat and mainly used as arable land. Due to scarce precipitation, the discharge of municipal wastewater treatment effluent contributes considerably to the total runoff, which can exceed the 50% ratio in dry seasons. Nodbach is an upstream tributary of the Wulka river. Its 76 km^2^ large catchment mostly consists of arable land on rather flat terrain and does not include any wastewater treatment plant. The catchment of the Raba river covers an area of approximately 1000 km^2^ and presents a more mixed land use of arable land, pastures, forests, and natural vegetation. Although multiple municipal and industrial wastewater treatment plants are present in the catchment, their total discharge contributes only to a low extent to the total river flow. Detailed hydrological and land use-related characteristics as well as the exact coordinates of the sampling sites at their outlets are reported in Table SI 1 of the Supplementary Information (SI).

All samples for the analysis in total water were taken via autosamplers as flow-composite samples, which covered the whole duration of the six events: at the Raba river via a Bühler 2000 portable automatic water sampler with 24 HDPE 1-l bottles stored in a cooled (~ 4 °C) compartment, at the Wulka and Nodbach using Endress + Hauser vacuum autosamplers with 24 1-l glass bottles stored at constant temperature of 4 °C. With respect to SPM, different approaches and devices were selected to collect, as far as possible in the same time of water sampling, sufficient representative material required for the PAHs analysis (30–60 g dm). During one event at the Wulka river, a grab sample was taken owing to the extremely high TSS level (1126 mg l^−1^) despite no flow increase, most likely induced by a very intensive and spatially localized storm. In the other cases, SPM was collected with Philipps samplers installed in the Wulka and in the Nodbach and with a Large Volume Sampler (LVS) operated at the Raba, respectively. Phillips samplers are devices developed by Phillips et al. (2000) for small catchments, which utilize ambient flow to induce sedimentation by settling. Despite their relative simplicity and low cost, they performed well in terms of sample representativity and comparability in a recent comparative assessment of four different sampling devices Keßler et al. (2020). The slightly adapted construction utilized in this study consists of 1-m-long PP pipes (DN 110), equipped with several reducers towards the outlet down to DN 40, to minimize the flow resistance. Further, the inlet opening is rotated by 90° in order to ensure sampling only during the targeted higher flow levels. A picture of the employed Phillips samplers is provided in Figure SI 1. The LVS is instead a more complex device conceived by Kittlaus and Fuchs (2015) to maximize the amount of SPM collected and its representativity of the actual SPM transported in rivers. It consists of a 1 m^3^ stainless steel tank, equipped with an optical level sensor which enables fully automated, event-based sampling. After the tank has been filled at the end of the sampled event, settling is allowed to take place in the tank for 2 to 3 days, after which the supernatant is either discharged or separately collected and the settled SPM is collected through the bottom drain in a 25-l glass vessel. A picture of the LVS operated at the Raba river is shown in Figure SI 2.

Table 1 provides a summary of the samples, sampling time, hydrological conditions during sampling, and employed sampling methods and devices. The events sampled in the study were characterized by mean TSS levels ranging from 256 to 1186 mg l^−1^, thus covering a broad spectrum of levels of riverine TSS transport. In addition to the already mentioned first sample from the Wulka river, in which the turbidity increased considerably without any rise in discharge, it can be observed that during the sampling in the Nodbach the turbidity increase was accompanied by only a modest rise compared to baseflow. As in the previous case, this event was also caused by a spatially localized storm. By contrast, the remaining four samples were taken at high-flow conditions.

**Table 1:** Overview of the samples taken in the study. *MQ*, long-term mean flow; *Q*_*mean*_, mean flow during sampling time; *TSS*_*mean*_, mean TSS concentration during sampling time; *LOI*, loss of ignition in the SPM samples

River	Water sampling					SPM sampling		
	MQ (m^3^ s^−1^)	*Q*_mean_ (m^3^ s^−1^)	TSS_mean_ (mg l^−1^)	Sampling time (UTC)	Sampling method/device	Sampling time (UTC)	Sampling method/device	LOI (% dm)
Wulka	1.12	0.72	1126	10.05.2022 09:15–10.05.2022 10:55	Flow-proportional composite via autosampler	10.05.2022 09:15–10.05.2022 15:15	Grab sample	14
Nodbach	0.09	0.12	531	14.09.2022 20:17–15.09.2022 03:10	Flow-proportional composite via autosampler	12.09.2022 12:45–15.09.2022 14:00	Phillips sampler	21
Raba	6.89	20.50	256	17.01.2023 06:15–18.01.2023 02:35	Flow-proportional composite via autosampler	17.01.2023 03:24–19.01.2023 01:46	Large Volume Sampler	15
Raba	6.89	46.88	1186	24.01.2023 06:30–24.01.2023 20:00	Flow-proportional composite via autosampler	24.01.2023 00:22–25.01.2023 07:30	Large Volume Sampler	11
Raba	6.89	44.20	944	14.04.2023 03:35–14.04.2023 16:00	Flow-proportional composite via autosampler	14.04.2023 02:19–14.04.2023 23:55	Large Volume Sampler	13
Wulka	1.12	8.80	375	14.04.2023 10:48–15.04.2023 08:48	Flow-proportional composite via autosampler	22.03.2023 14:40–17.04.2023 17:00	Phillips sampler	11

### Sample preparation

Water samples were transported and stored at 2–6 °C and were partly filtered with a vacuum filtration device using a 0.7-μm glass fiber filter. Filtered and unfiltered water samples were filled into 1-l amber glass bottles and immediately stabilized with n-hexane. SPM samples were decanted on site if taken with LVS or in the laboratory if taken either with Phillips samplers or as grab samples. The decanted SPM was frozen at − 20 °C and subsequently lyophilized with sublimator VaCo 2 from Zirbus. Prior to the chemical analyses, the material was sieved in order to retain only particles with a size below 2 mm. For the samples collected with LVS, the supernatant was retained, filled into 1-l amber glass bottles and immediately stabilized with n-hexane. All bottles and larger glass containers for water and SPM samples as well as the measuring and auxiliary vessels were cleaned with spirit, tap water, deionized water, and acetone prior to each use.

### Chemical analyses

Eight PAH substances were included in the study. The selection focused on PAHs with high tendency to adsorption to suspended solids in water due to high n-octanol/water partition coefficients (log Kow) and low solubility, and thus for which the concentrations of the particle-bound fraction are expected to become dominant at higher TSS content in total water samples. The selected PAHs are as follows: Benzo(a)pyrene (BaP), Benzo(a)anthracene (BaA), Benzo(b)fluoranthene (BbF), Benzo(g,h,i)perylene (BghiP), Chrysene (Chry), Fluoranthene (Fla), Indeno(1,2,3-c,d)pyrene (Ind123cdP), and Pyrene (Pyr). Detailed information on molar mass, log Kow, and water solubility is given in Table SI 2.

PAH analyses in both water (DIN 38407–39) and SPM samples (EN 15527:2008–07) were carried out by an accredited laboratory. The standard method DIN 38407–39 is routinely applied in the Austrian national official monitoring. In accordance with this standard, the sample should be extracted within 24 h. Alternatively, if 25 ml of the extraction agent is added immediately upon sampling, the sample can be stored in the dark at 4–8 °C for 72 h until processing. In this study, n-hexane was added directly after sampling. In the case of water samples, the addition of deuterated surrogate standards (in accordance with DIN 38407–39, at least three internal standards are required; however, in this work specific surrogates were added for all the investigated PAH substances) was followed by a liquid–liquid extraction with n-hexane and stirring for 1 h. The stirring process was conducted in a manner that ensured the formation of a stirring funnel extending to the bottom of the vessel. The exact volume used was determined by differential weighing of the sample bottle. In lyophilized SPM samples, after addition of eight deuterated standards, PAHs were extracted by Soxhlet extraction using a 1:1 mixture of hexane/acetone as solvent. Approximately 200 ml of the solvent mixture was added to 10–25 g of sample, which was then extracted for at least 8 h. The extract was transferred to a separating funnel, the acetone was removed by shaking twice with 400 ml of Milli-Q-water and the extract was dried with anhydrous sodium sulfate. For both types of samples, the extract was evaporated and concentrated with iso-octane as keeper, while the residue was dissolved in iso-octane. At the end of the sample preparation procedure, an injection standard was added. The PAH analyses were performed with gas chromatography-mass spectrometry (GC–MS). The quantification was performed with the standard method of recovery rate correction, based on the initially added deuterated surrogate standards. The limits of quantification (LOQ) vary between 0.001 and 0.002 µg l^−1^ in the water matrix and between 3.6 and 8.3 µg kg^−1^ dm in SPM (detailed LOQ and limits of detection (LOD) for each specific PAH are reported in Table SI 3). Relatively high recovery rates were achieved in SPM — from the lowest range of 66–80% for BghiP to the highest one of 92–114 for BaP. In contrast, the water matrix presents lower recovery rates, with the lowest range of 42–68% for BaP and the highest one of 62–79 for Pyr. Specific recovery rates as well as analytical uncertainty for all PAHs in the different matrices are provided in Table SI 4.

Further, loss on ignition (LOI) was analyzed in the SPM samples according to the standard method ÖNORM EN 12879, while TSS was analyzed in water samples following the standard method DIN 38409–2.

### Settling performance of suspended solids

An important reason to separately analyze the supernatant of the LVS was to control the settling performance of the suspended solids and thus to identify a potential falsification of the survey design and of the actual PAH content measured in the SPM. The comparison of the TSS content of bulk water samples with that of the corresponding supernatant samples indicates a settling performance above 95%, with exception of the sample with highest TSS content of 1186 mg l^−1^. In this case, the TSS content of the supernatant was 145 mg l^−1^, which denotes a relatively poorer but still satisfactory settling performance of 88%.

### Estimation of PAH concentration in bulk water from content in SPM

Theoretical PAH concentrations in bulk water were derived combining the PAH measured values in SPM with the TSS content of bulk water and with the PAH measured values in filtered water via Eq. 1:

1 $${C}_{w,tot}={C}_{SPM}\bullet {TSS}_{w} +{C}_{w,dis}$$

where *C*_*w,tot*_ denotes the estimated total PAH aqueous concentration, *C*_*SPM*_ the measured PAH concentration in SPM, *TSS*_*w*_ the TSS content of the bulk water samples, and *C*_*w,dis*_ the measured PAH concentration in the filtered fraction of the water samples. The analytical uncertainty reported in Table SI 4 was explicitly considered in this calculation by applying the Gaussian principles of error propagation. Equation 1 relies on the assumption that there is no significant difference between the PAH concentration in SPM samples and the PAH content adsorbed to the TSS in their corresponding bulk water samples, respectively. The implications of such assumption for the results interpretation are discussed in the next section.

## Results and discussion

### Overview of the measurements in all matrices

The experimental setup successfully delivered sufficient amounts of SPM simultaneously to bulk water samples for each of the turbidity events investigated. This allowed PAH measurements to be carried out in all matrices as planned. The complete measurement results are reported in Table 2. Fla and Pyr, the two PAHs with the lowest molecular weight among the selected ones, were the only ones being detected in the filtered water samples, where they were present in the concentration range of 1–7 ng l^−1^. The very same pattern was identified in two supernatant samples. In the third one, also BaP was quantified at 2 ng l^–1^ and Fla and Pyr were detected at higher concentrations of 4–5 ng l^−1^. This supernatant sample corresponds to the SPM sample with lower settling performance. Thus, the difference can be explained by the higher content of fine particles in suspension. Further, Fla and Pyr are the two PAHs which consistently show the highest values in each bulk water and SPM sample. In bulk water, they occurred with a median value of 24 ng l^−1^ and with maximum values reaching 49 and 77 ng l^−1^, respectively. In SPM, their median concentration levels were 43 and 37 µg kg^_1^ dm, with maximum peaks of 210 and 170 µg kg^−1^ dm, respectively. The other six PAHs with higher molecular weight can be regarded as a separate homogenous group, owing to a consistent and similar pattern of occurrence and concentration levels. In SPM they were always above LOQ, with median values ranging from 20 µg kg^−1^ dm for Ind123cdP to 26 µg kg^−1^ dm for BaA. In the direct analysis of two bulk water samples, by contrast, some of these PAHs were detected below LOQ, while the rest was quantified with values ranging from 2 to 22 ng l^−1^. The fact that Fla and Pyr were found in both matrices in consistently higher concentrations than the remaining higher molecular weight PAHs matches with the same pattern observed by Chiffre et al. (2015). Also Nagy et al. (2014) identified Fla and Pyr as the two dominant PAH substances in the sediments of the Danube River and its tributaries in Hungary. The PAH levels measured in SPM are consistent with the ones found in the same rivers in previous works (Zoboli et al. 2019; Jolankai et al. 2022) and they confirm the observed higher particle-bound transport of PAHs in the strongly agricultural Wulka catchment (including the tributary Nodbach) compared to the Raba catchment, which is characterized by a more mixed land use with a greater share of grassland and forests. Moreover, these results but also the measured values in bulk and filtered water support what was already observed by Zoboli et al. (2019), namely that the PAHs contamination in these rivers falls at the lower end of the broad range of PAH contamination levels published in the international literature, which has largely focused on more heavily urbanized or industrialized river catchments (Chiffre et al. 2015; Le Meur et al. 2017; Wölz et al. 2010; Abuhelou et al. 2017).

**Table 2:** Results of the PAH measurements in the different sample matrices. *nd*, not detected (< LOD); *nq*, not quantified (< LOQ)

River	Date	PAH	Bulk sample (µg l^−1^)	Filtered sample (µg l^−1^)	Supernatant (µg l^−1^)	SPM (µg kg^−1^ dm)
Wulka	10.05.2022	Fla	0.025	0.0015	-	42
		Pyr	0.027	0.0023	-	37
		BaA	0.0095	nd	-	16
		Chry	0.011	nd	-	23
		BbF	0.012	nd	-	25
		BaP	0.011	nd	-	24
		BghiP	0.0079	nd	-	24
		Ind123cdP	0.0065	nd	-	19
Nodbach	14.09.2022–15.09.2022	Fla	0.049	0.004	-	130
		Pyr	0.077	0.0068	-	130
		BaA	0.020	nd	-	37
		Chry	0.019	nd	-	71
		BbF	0.021	nd	-	60
		BaP	0.022	nd	-	59
		BghiP	0.017	nd	-	73
		Ind123cdP	0.014	nd	-	53
Raba	17.01.2023–18.01.2023	Fla	0.0027	0.0019	0.0025	44
		Pyr	0.0025	nd	0.0023	36
		BaA	nq	nd	nd	18
		Chry	nq	nd	nd	15
		BbF	nq	nd	nd	21
		BaP	0.0017	nd	nd	21
		BghiP	nq	nd	nd	21
		Ind123cdP	nq	nd	nd	16
Raba	24.01.2023	Fla	0.024	0.0014	0.005	34
		Pyr	0.021	nq	0.0044	27
		BaA	0.0081	nd	nq	14
		Chry	0.012	nd	nq	11
		BbF	0.0079	nd	nq	14
		BaP	0.0078	nd	0.002	15
		BghiP	0.0064	nd	nq	15
		Ind123cdP	0.0047	nd	nq	12
Raba	14.04.2023	Fla	0.012	nq	0.0018	36
		Pyr	0.01	nd	0.0014	29
		BaA	0.0036	nd	nd	13
		Chry	0.0036	nd	nd	17
		BbF	0.0027	nd	nd	18
		BaP	0.0037	nd	nd	15
		BghiP	nq	nd	nd	16
		Ind123cdP	0.0021	nd	nd	11
Wulka	14.04.2023–15.04.2023	Fla	0.023	0.001	-	210
		Pyr	0.028	0.0023	-	170
		BaA	0.008	nd	-	85
		Chry	0.0082	nd	-	87
		BbF	0.0061	nd	-	82
		BaP	0.0076	nd	-	68
		BghiP	0.0049	nd	-	63
		Ind123cdP	0.0045	nd	-	54

**Fig. 1:**
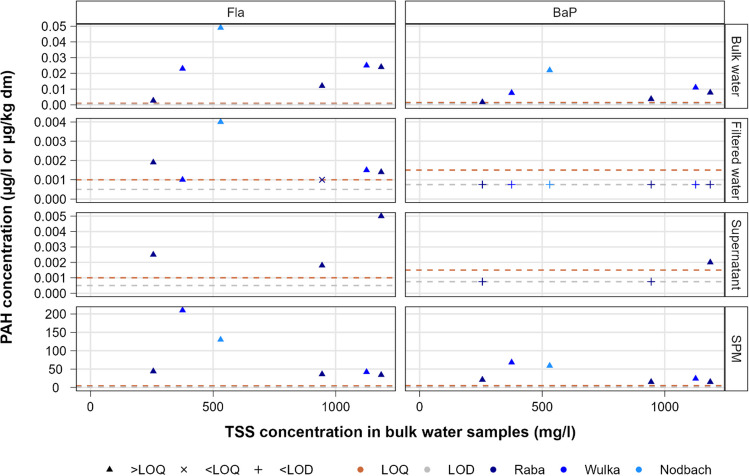
Overview of Fla and BaP concentrations in the four analyzed matrices; nq values are depicted equal to LOQ and nd values as LOD, respectively

Figure 1 depicts illustratively for Fla and BaP the results of the measurements in the four matrices against the TSS content in the water samples. In Figure SI 3, which shows the results for all PAHs, it is clearly visible that Pyr follows the same pattern as Fla, while the other five substances exhibit the pattern identified for BaP. In line with theoretical expectations deriving from their hydrophobic nature and adsorption affinity, bulk water samples show a tendency towards higher concentrations of all selected PAHs at higher TSS levels. This is consistent with the findings of previous studies, such as the positive linear correlation between PAHs concentrations in bulk water samples and turbidity observed in German river catchments by Rügner et al. (2013), and the positive relationship between PAHs concentrations in the estuarine water phase and the amount of transported particulate matter identified by Niu et al. (2018). The extent of such differences has the potential to play a relevant role for the robust assessment of compliance with EQS, in case such evaluation is based on the water matrix instead of biota. For example, the annual average EQS (AA-EQS) value of 6.3 ng l^−1^ established for Fla was exceeded in all bulk water samples, except in the one with the lowest average TSS content of 256 mg l^−1^, while the measured values in filtered samples, which can be expected to be similar to the concentrations at baseflow conditions in absence of rainfall events, were consistently below it. With respect to SPM, the measured PAH values in the Raba river exhibit a stable concentration level over a large TSS spectrum. The same level was measured in the Wulka sample with highest TSS. By contrast, higher PAH values were detected in the Nodbach and in the Wulka sample with lower TSS content. In the case of the Nodbach, the reason may partly be found in the relatively higher organic matter content of the SPM sample. In this respect, Moeckel et al. (2014) revealed that the concentration of PAH substances with five or more aromatic rings in streams draining organically rich soils was strongly correlated to the concentration of dissolved organic matter (DOC) and that the PAHs with four and more rings had a similar seasonal pattern as DOC. Moreover, Niu et al. (2018) found that particulate organic matter was one of the main factors determining the distribution of PAHs in SPM. By contrast, for the Wulka sample, the explanation may lie in the mobilization of locally more contaminated sediments or in a potentially greater share of fine particles with larger adsorption surface.

### Comparison between measured and theoretical concentrations

**Fig. 2:**
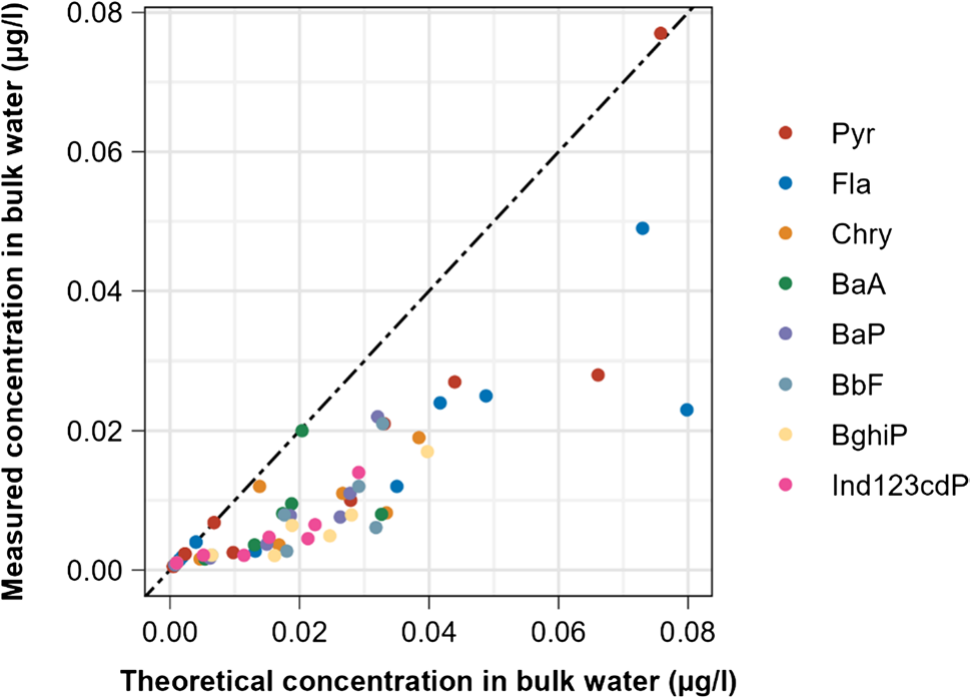
Comparison of the PAH concentrations analyzed in bulk water samples with those theoretically derived using Eq. 1. The dotted line depicts the perfect correspondence between measured and calculated values

**Fig. 3:**
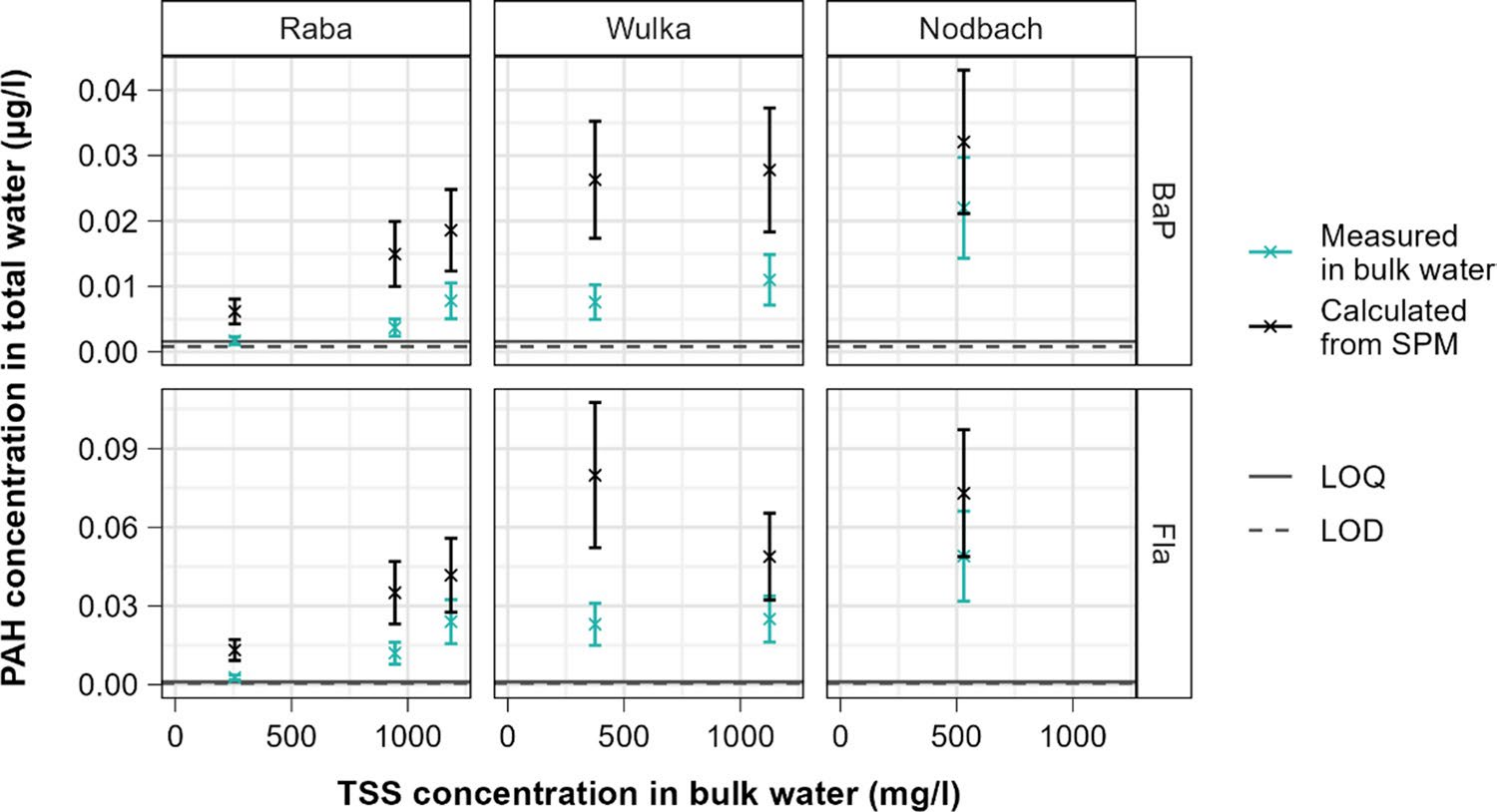
Comparison of Fla and BaP concentrations analyzed in bulk water samples with those theoretically derived using Eq. 1 depicted with their specific uncertainty

The comparison between concentrations measured in bulk water samples and theoretical concentrations expected for the same samples according to Eq. 1 is shown for all PAHs in Fig. 2. Figure 3 illustrates exemplarily for Fla and BaP the same comparison more in detail and with inclusion of the specific uncertainties. The results support the hypothesis of a systematic underestimation of the real concentrations through the analysis of water samples with elevated TSS content. Despite a relatively high degree of variability, the vast majority of the measured values deviates substantially from the theoretical ones, with all detected deviations consistently pointing to an underestimation of real total concentrations. In average, the direct analysis in water determined about 40% of the theoretical total concentration. Table 3 indicates the specific ratios for all considered PAHs, which vary between 31 and 55% and thus correspond well with the 40–60% range reported by Busch et al. (2007). It is interesting to observe that the calculated mean ratios suggest the existence of a rising trend of deviation with increasing molecular weight and adsorption affinity. Although the variance in the relatively small dataset does not allow considering such trend as statistically significant, it depicts a plausible pattern. As previously mentioned, there is multiple published evidence of the analytical problems related to the incomplete extraction of PAHs adsorbed to suspended solids in the preparatory step prior to the analysis of aqueous samples. It can be thus reasonably expected that the incomplete extraction is more pronounced the stronger PAH substances are adsorbed to solids. This is consistent with the findings of Brum et al. (2008) research, which used principal component analysis to cluster PAH substances based on their optimal extraction conditions. Their results showed that the primary factor driving the grouping was molecular weight.

**Table 3:** Average ratio of PAH concentrations measured in bulk water samples to those theoretically derived from measurements conducted in SPM (mean value and standard deviation calculated from six samples, no unit)

Fla	Pyr	BaA	Chry	BbF	BaP	BghiP	Ind123cdP
0.43 ± 0.18	0.55 ± 0.27	0.46 ± 0.28	0.43 ± 0.24	0.36 ± 0.18	0.39 ± 0.16	0.28 ± 0.11	0.31 ± 0.11

The liquid–liquid extraction procedure used in this study is a standard and well-established method. It is considered a good compromise as it is simple and fast and delivers plausible and reliable results. Nevertheless, it is apparent that the method would benefit from optimization when handling water samples with elevated TSS content. Insufficient contact time with the solvent could be a serious constraint and therefore an improvement could be achieved by increasing the extraction time beyond the 1-h duration employed in this study. In this respect, Brum et al. (2008) achieved high recovery rates in river water samples with an experimental setting of liquid–liquid extraction involving a total volume of 77 ml of hexane, divided into four extraction steps of 18 min each. Further, they indicated that a larger volume of solvent is necessary for the extraction of the heaviest PAHs. Additionally, the performance of an alternative solvent to n-hexane could be tested. Although the scope of the analyzed matrix did not specifically address the issue of samples with high TSS content, Yan et al. (2018) compared different variants of a simplified liquid–liquid extraction method. They found that the best extraction performance for surface water was obtained using a mixture of acetonitrile and dichloromethane. However, acetonitrile is miscible with water, which prevents phase separation. In addition to n-hexane, the DIN 38407–39 standard proposes the following solvents for extraction: isohexane, heptane, petroleum ether (40/60), dichloromethane, toluene. It should be noted that dichloromethane often contains stabilizers, e.g., ethanol or pentane, which can influence the elution power of the eluent. If the stabilizer is missing or removed, the formation of radicals is to be expected, which can lead to reduced findings of individual PAHs. The presence of hydrogen chloride indicates radicals and can be detected after shaking dichloromethane with water and by measuring the pH.

Considering the analytical uncertainty in the comparison (Fig. 3) leads to a partial overlap of the measured and calculated concentrations for one sample in the case of BaP and for three samples in the case of Fla, although of considerable extent only for the sample taken in the Nodbach. For the majority of samples in the survey, the conclusion of a consistent underestimation is not affected by the consideration of this source of uncertainty. The interpretation of the results shall consider additional factors, which bring a certain degree of uncertainty in the study. In order to collect sufficient quantities of SPM required for the chemical analysis of PAHs, water and SPM had to be sampled separately and over slightly different periods of time. The resulting differences in composition and representativeness of the two different types of samples may somewhat affect the comparison between the PAH concentrations directly measured in the bulk water samples with those calculated from the analysis in SPM. Nevertheless, this source of uncertainty is not expected to bring a systematic bias in the results, but rather a random error. Thus, the high consistency coupled with the large extent of deviations identified between the different methodological approaches is such to allow drawing clear conclusions. Further, although best available techniques and devices were applied for the collection of SPM, capturing the finest particles transported in rivers remains challenging due to their resistance to settling. Accounting for this potential error would however lead to calculating even higher theoretical PAH concentrations in bulk water, given the large specific surface provided by such particles for the adsorption of organic contaminants. It would therefore further strengthen the conclusions reached in the study.

## Conclusions

The targeted survey based on simultaneous time-integrated sampling of different river matrices during six high-turbidity events reveals that a strategy based on the analysis of bulk water samples under conditions of increased suspended sediment transport can lead to a systematic and considerable underestimation of PAH concentrations. This finding has multiple consequences for scientists and water authorities. Studies which investigate the dynamics and the behavior of particulate PAH transport in river systems relying on regression models based on PAH and TSS analyses in bulk water samples are exposed to a systematic negative bias. Official monitoring programs consisting of 12 bulk water samples in a year bear the risk of false non-detects and of failing in identifying potential exceedances of EQS for PAHs. The likely underestimation of real PAHs concentrations due to analytical problems in aqueous samples with high TSS content is further exacerbated by the fact that most often conditions of elevated TSS transport are underrepresented in official surveys. In this respect, the improvement of the chemical analyses alone would not necessarily make the assessment more representative, as long as the sampling is performed without considering TSS dynamics at sampling dates and during the whole year. In an opposite scenario with overrepresentation of samples with high TSS content and enhanced analytical performance, EQS exceedance and transported riverine loads could potentially be overestimated. Whereas a more representative and robust alternative for the assessment of compliance with EQS for PAHs is thus the measurement in biota, this approach would offer neither a useful data basis nor any improvement for (i) studying and understanding the transport dynamics of PAHs in river systems and (ii) estimating reliable PAHs riverine loads required to validate emission and water quality models, which in turn are essential tools for risk assessment and for the generation of emission inventories. In order to ensure a solid data basis for the latter two objectives, parallel efforts aimed to (i) improve the chemical analyses in bulk water samples with elevated TSS content (e.g., with improved extraction methods or with complementary extraction and analysis of filtered water matrix and of filters), (ii) introduce complementary monitoring surveys for the separate collection of SPM, and (iii) complement and improve the sampling of bulk water with the support of continuous TSS monitoring are deemed necessary.

### Supplementary Information

Below is the link to the electronic supplementary material.

Supplementary file1 (DOCX 1271 KB)

## Data Availability

Data are available upon request.
