# Supplementary material for: Systematic underestimation of polycyclic aromatic hydrocarbon aqueous concentrations in rivers
Source: Environ Sci Pollut Res Int. 2024 May 25;31(26):38117–27. doi: 10.1007/s11356-024-33787-9 (PMC11189336; doi:10.1007/s11356-024-33787-9)
Supplement: Supplementary file 1 — Supplementary file1 (DOCX 1271 KB) [file 11356_2024_33787_MOESM1_ESM.docx]

**SUPPLEMENTARY INFORMATION**

**Systematic underestimation of polycyclic aromatic hydrocarbons aqueous concentrations in rivers**

Ottavia Zoboli^1*^, Nikolaus Weber^1^, Katharina Braun^2^, Jörg Krampe^1^, Matthias Zessner^1^

^1^ Institute for Water Quality and Resource Management, TU Wien, Karlsplatz 13, 1040 Vienna, Austria

^2^ Environment Agency Austria, Spittelauer Lände 5, 1090 Vienna, Austria

* Corresponding author

Table of content

[Sampling 2](#_Toc144812142)

[Chemical analyses 3](#_Toc144812143)

[Results 5](#_Toc144812144)

# Sampling

Table SI 1: Main characteristics of the catchments of the three sampling locations included in the study.

|  | Wulka | Nodbach* | Raba |
| --- | --- | --- | --- |
| Sampling point (outlet) coordinates | 47°51'03.5" N 16°37'49.2" E | 47°48'46.6" N 16°36'07.6" E | 46^°^55′48′′ N 16^°^9′12′′ E |
| Catchment area (km^2^) | 389 | 76 | 1009 |
| Mean annual flow (m^3^ y^-1^) | 1.12 | 0.09 | 6.89 |
| Mean annual precipitation (mm y^-1^) | 695 | 640 | 833 |
| Share of wastewater (municipal and industrial) on river MQ at catchment outlet (%) | 36 | 0 | 3 |
| Land use | | | |
| Arable land (%) | 50 | 61 | 25 |
| Pastures (%) | 2 | 3 | 17 |
| Forests and natural vegetation (%) | 40 | 30 | 52 |
| Urban areas (%) | 5 | 5 | 3 |
| Other land use (%) | 3 | 1 | 3 |

*subcatchment of the Wulka river


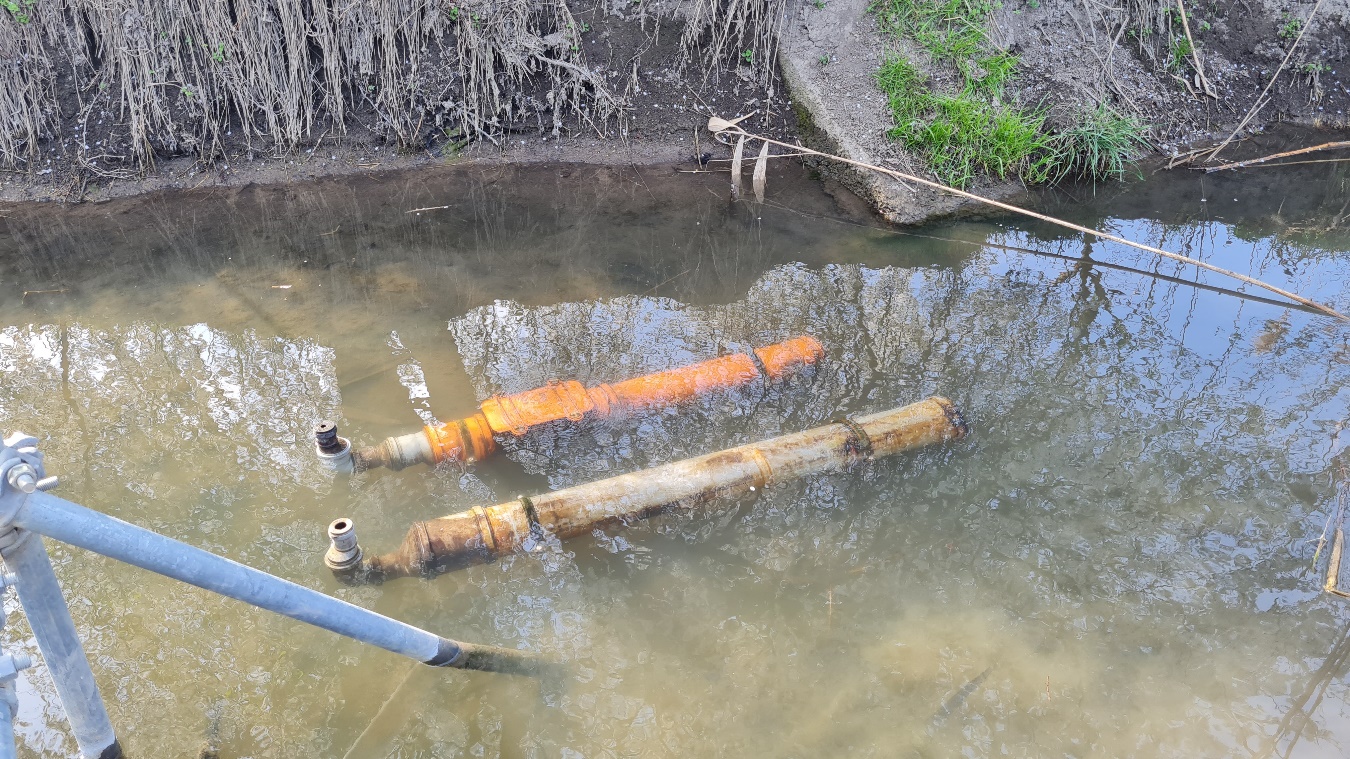


Figure SI 1: Phillips samplers employed in the study.


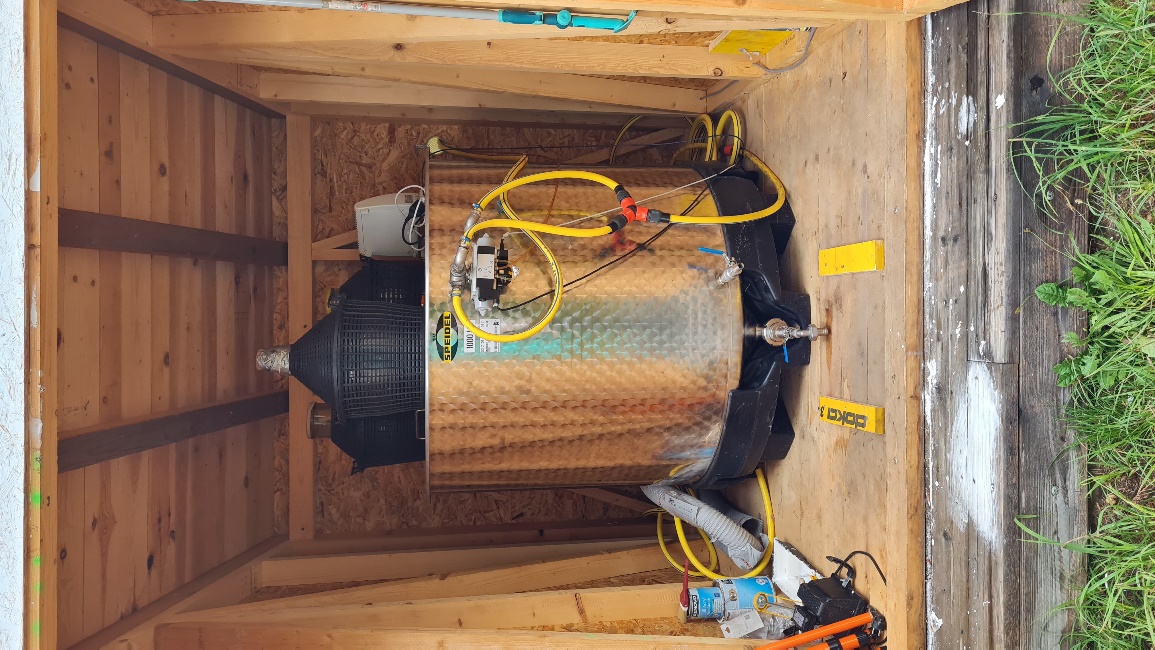

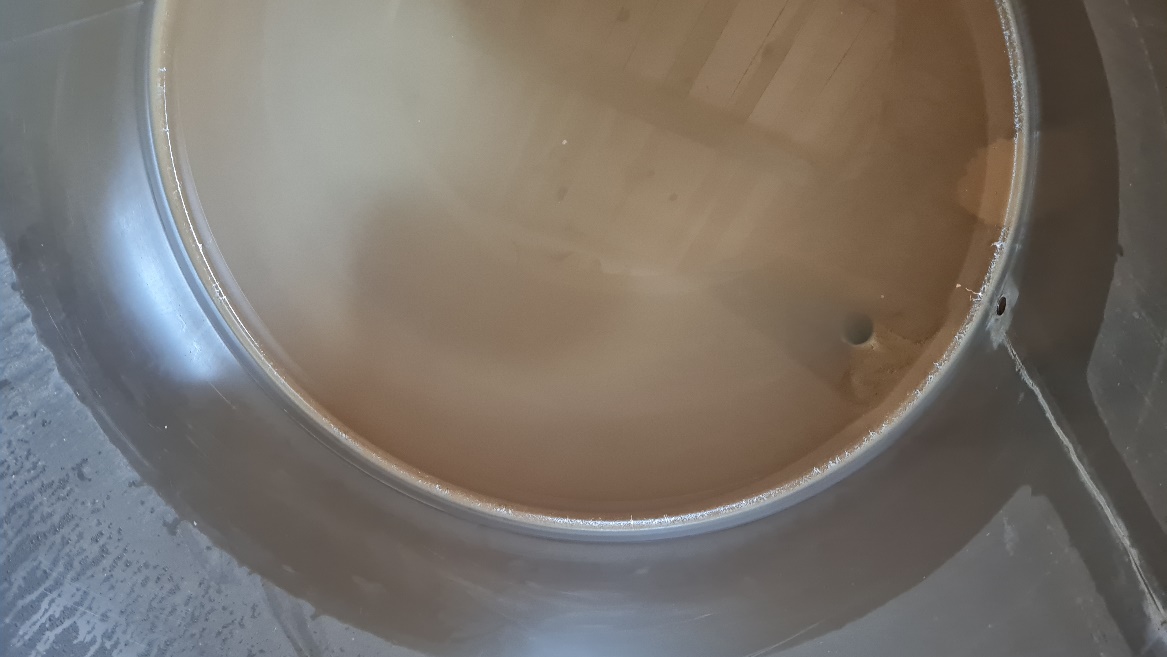


Figure SI 2: Left: Large Volume Sampler in operation at the Raba water quality monitoring station, constructed after the design of Kittlaus and Fuchs (2015); Right: settled SPM at the bottom of the SLV after a resting time of two days after finishing the automated sampling.

# Chemical analyses

Table SI 2: Selected PAHs included in the study. Data obtained from: PubChem database (https://pubchem.ncbi.nlm.nih.gov/)

| Parameter | Abbreviation | Molar mass (g mol^-1^) | log KOW | Water solubility (mg l^-1^) at 25°C |
| --- | --- | --- | --- | --- |
| Pyrene | Pyr | 202 | 4.88 | 0.13 |
| Fluoranthene | Fla | 202 | 5.16 | 0.23 |
| Chrysene | Chry | 228 | 5.73 | 0.002 |
| Benzo(a)anthracene | BaA | 228 | 5.79 | 0.009 |
| Benzo(a)pyrene | BaP | 252 | 6.13 | 0.0016 |
| Benzo(b)fluoranthene | BbF | 252 | 5.78 | 0.0015 |
| Benzo(g,h,i)perylene | BghiP | 276 | 6.63 | 0.0003 |
| Indeno(1,2,3-c,d)pyrene | Ind123cdP | 276 | 6.76 | 0.0007 |

Table SI 3: Limit of quantification (LOQ) and limit of detection (LOD) of the PAH analyses carried out in the study for the matrix water and SPM, respectively.

| Parameter | Matrix | LOQ | LOD | Unit |
| --- | --- | --- | --- | --- |
| Pyrene | Water | 0.001 | 0.0005 | µg l^-1^ |
| Fluoranthene | Water | 0.002 | 0.001 | µg l^-1^ |
| Chrysene | Water | 0.002 | 0.001 | µg l^-1^ |
| Benzo(a)anthracene | Water | 0.0012 | 0.0006 | µg l^-1^ |
| Benzo(a)pyrene | Water | 0.001 | 0.0005 | µg l^-1^ |
| Benzo(b)fluoranthene | Water | 0.0011 | 0.00055 | µg l^-1^ |
| Benzo(g,h,i)perylene | Water | 0.0015 | 0.00075 | µg l^-1^ |
| Indeno(1,2,3-c,d)pyrene | Water | 0.0015 | 0.00075 | µg l^-1^ |
| Pyrene | SPM | 4 | 1.2 | µg kg^-1^ dm |
| Fluoranthene | SPM | 4.3 | 1.2 | µg kg^-1^ dm |
| Chrysene | SPM | 6.6 | 1.9 | µg kg^-1^ dm |
| Benzo(a)anthracene | SPM | 7.5 | 2.3 | µg kg^-1^ dm |
| Benzo(a)pyrene | SPM | 4.8 | 1.2 | µg kg^-1^ dm |
| Benzo(b)fluoranthene | SPM | 8.3 | 2.5 | µg kg^-1^ dm |
| Benzo(g,h,i)perylene | SPM | 6.6 | 2 | µg kg^-1^ dm |
| Indeno(1,2,3-c,d)pyrene | SPM | 3.6 | 1 | µg kg^-1^ dm |
|  |  |  |  |  |

Table SI 4: Recovery rates achieved in the PAH analyses performed in this study for water matrix and SPM matrix, respectively and uncertainty of the measurements.

| Parameter | Matrix | Recovery rate (%) | Uncertainty (%) |
| --- | --- | --- | --- |
| Pyrene | Water | 62-79 | 25 |
| Fluoranthene | Water | 60-79 | 25 |
| Chrysene | Water | 63-66 | 25 |
| Benzo(a)anthracene | Water | 66-69 | 25 |
| Benzo(b)fluoranthene | Water | 61-65 | 25 |
| Benzo(a)pyrene | Water | 42-68 | 35 |
| Benzo(g,h,i)perylene | Water | 58-60 | 35 |
| Indeno(1,2,3-c,d)pyrene | Water | 58-61 | 35 |
| Pyrene | SPM | 73-82 | 30 |
| Fluoranthene | SPM | 70-82 | 25 |
| Chrysene | SPM | 86-105 | 25 |
| Benzo(a)anthracene | SPM | 88-98 | 25 |
| Benzo(b)fluoranthene | SPM | 91-112 | 30 |
| Benzo(a)pyrene | SPM | 92-114 | 30 |
| Benzo(g,h,i)perylene | SPM | 66-80 | 30 |
| Indeno(1,2,3-c,d)pyrene | SPM | 70-87 | 30 |

# Results


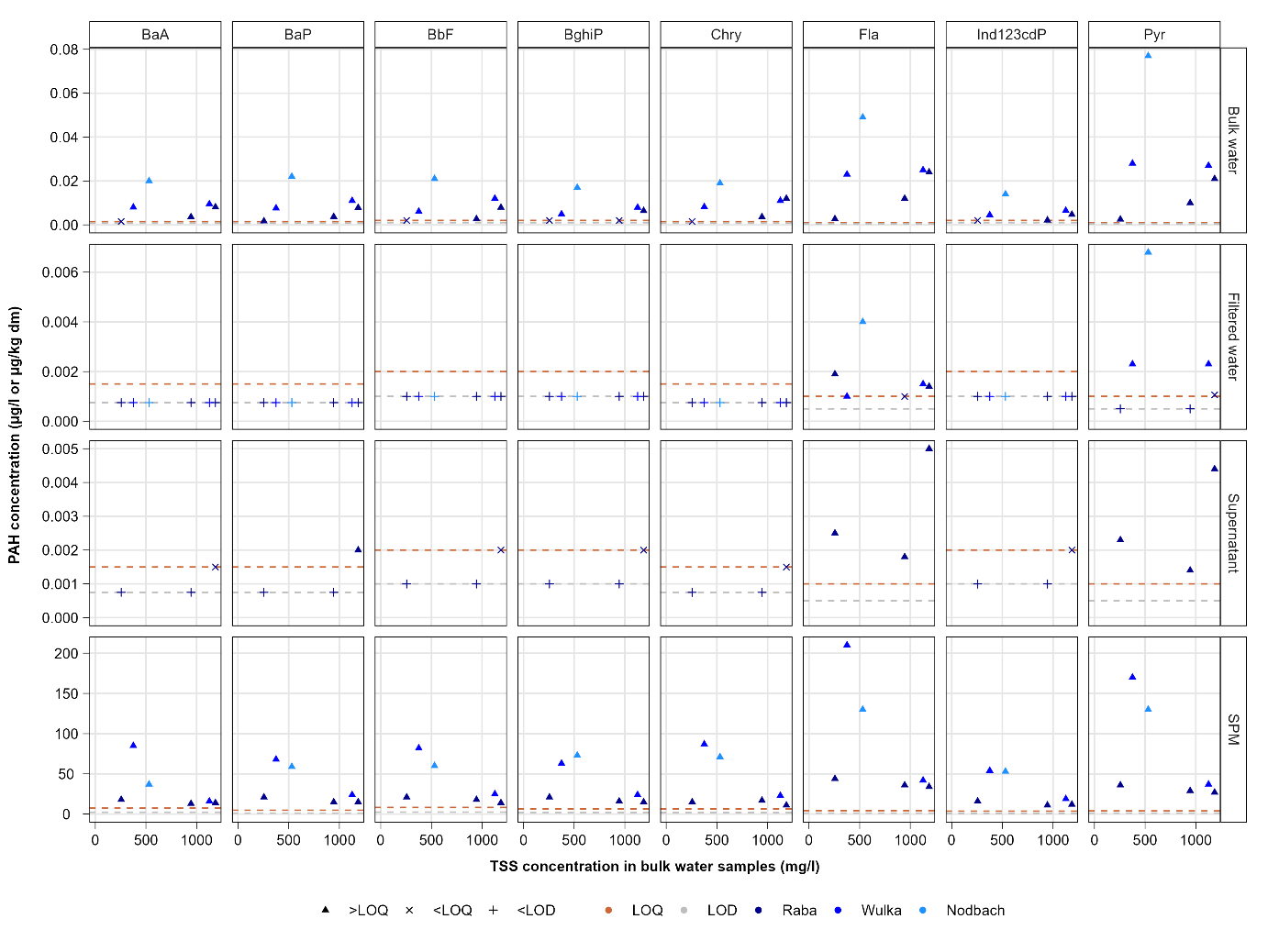


Figure SI 3: Overview of the concentrations of all 8 measured PAHs in the four analysed matrices; nq values are depicted equal to LOQ and nd values as LOD, respectively.
